# Ectopic cutaneous *Schistosoma haematobium* in the inguinal region

**DOI:** 10.1093/jtm/taz011

**Published:** 2019-03-07

**Authors:** Lennert Slobbe, Thomas B J Demeyere, Perry J J van Genderen, Cathy C Simon, Willem J Huisman

**Affiliations:** 1 Department of Medical Microbiology and Infectious Diseases, Erasmus MC University Hospital, Rotterdam, The Netherlands; 2 Department of Pathology, PAMM Foundation Eindhoven, Eindhoven, The Netherlands; 3 Department of Internal Medicine, IJsselland Ziekenhuis, Capelle aan den IJssel, The Netherlands; 4 Department of Dermatology, St. Anna Hospital, Geldrop, The Netherlands

## Abstract

A 66-year-old man with indolent systemic mastocytosis presented with a plaque-like cutaneous lesion at his right inguinal region. He had travelled to various African countries in the years before. Pathological examination revealed a granulomatous infiltrate surrounding eggs of *Schistosoma haematobium*. Ectopic cutaneous schistosomiasis was therefore diagnosed, for which he was treated with Praziquantel.

A 66-year-old man with indolent systemic mastocytosis treated with antihistamine drugs presented with a sharply demarcated, erythematous, plaque-like lesion with a smaller satellite lesion located at his right inguinal region (Figure 1). The lesions had evolved during the last 3 months and had started as erythematous and infiltrated areas. The size gradually increased, with tenderness during the last few weeks. Empirically started topical antimycotics by the general practitioner had no result, after which he was referred to a dermatologist in January 2018. There were no systemic symptoms. A secondary bacterial infection of a psoriasis lesion was initially considered, but no response was observed with topical steroids. Pathological examination of a skin biopsy revealed an extensive dermal granulomatous inflammatory infiltrate composed of lymphocytes, plasmocytes and eosinophilic granulocytes surrounding parasite eggs with an apical spine (Figure 2), which were determined as *Schistosoma haematobium*. No adult worms were detected. Upon further request, he confirmed about his travelling to various African countries in the years before. In 2001, he had visited Zimbabwe and Botswana; in 2009, he had travelled to Namibia; in 2011, he went to Uganda and Kenia; in 2013, to Ethiopia; in 2015, to Zimbabwe, Malawi, Mozambique and Zambia; and finally, in 2017, he had travelled around South Africa. He also mentioned about swimming in freshwater in at least Malawi and Zimbabwe. Schistosoma serology, including both the enzyme-linked immunosorbent assay (ELISA) IgG for *Schistosoma* egg-antigen [value 800; reference, <222] and the indirect haemagglutination (IHA) test for adult worm-antigen [160; reference, <80], was positive. The eosinophil blood count was 0.12 × 10^9^/L [reference, 0.04–0.60 × 10^9^/L]. Additional analysis of urine or stool examination was not performed. Retrospectively, this would have been interesting, but testing has been left behind because the applied treatment with Praziquantel would not have differed with outcome. After two doses of praziquantel 40 mg/kg on days 0 and 28, regression of the lesions was noted.

**Figure 1.**
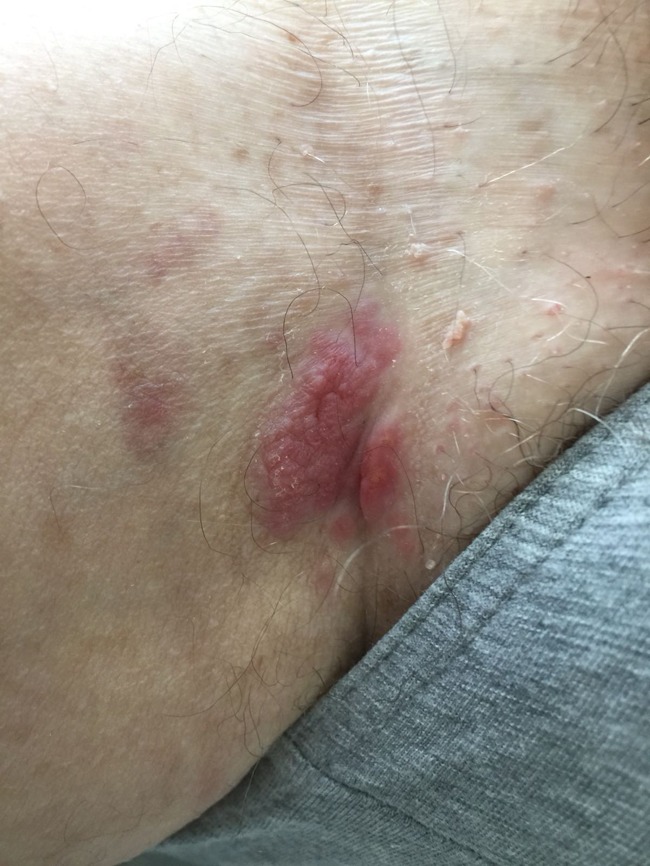
Inguinal schistosoma lesion.

**Figure 2.**
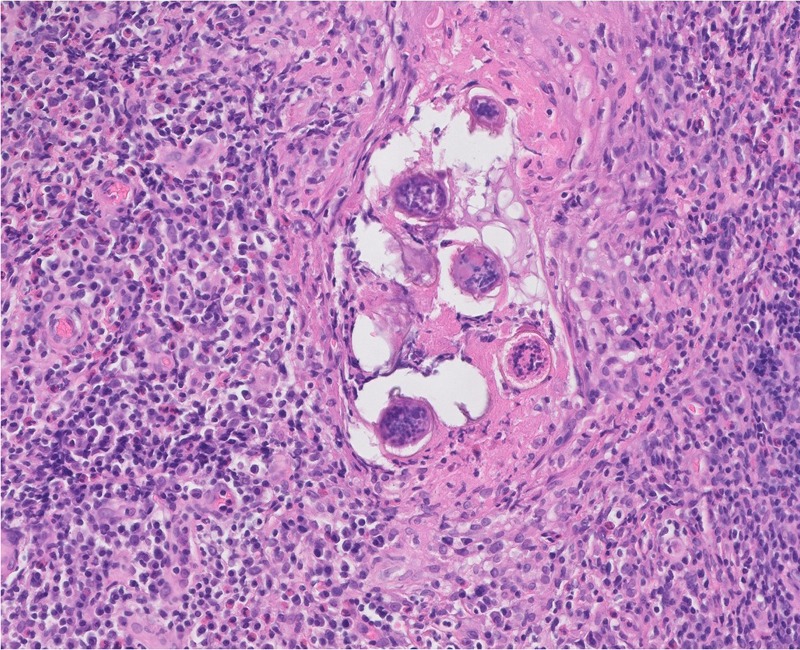
Eggs of *S. haematobium* in a skin biopsy specimen, stained with haematoxylin and eosin (H&E; 400× magnification).

The cutaneous disease manifestation as described in our case is a late disease manifestation and is completely different from cutaneous manifestations of early schistosomiasis. The latter may or may not occur after cercarial penetration of the skin and is described after infection with any schistosomal species, though some believe most prominently in case of *S. japonicum*.^1^ Ectopic cutaneous schistosomiasis is uncommon, the pathogenesis is not fully elucidated and a species predominance is not known.^2–5^ Patients become infected after the infectious cercariae, living in freshwater, penetrate the skin and enter the circulation. For cutaneous lesions in the anogenital and inguinal areas, it is hypothesized that venous or portosystemic anastomoses between the urinary bladder wall and the skin may enable venous spreading of eggs, especially at times of raised intra-abdominal pressure. Ectopic cutaneous disease outside the anogenital region is still even more rare and may depend on anastomoses between pelvic veins and the valveless vertebral venous plexus (Batson’s veins). This may enable eggs to reach the spinal circulation for further distribution and could explain the zosteriform distribution in case of such ectopic lesions. Sometimes, aberrant migration of adult worms may occur as copulating, egg-laying worms have been detected in skin biopsies before.

Ectopic cutaneous schistosomiasis is not often seen in travel medicine and could easily be misdiagnosed, which may cause a significant delay in time before appropriate therapy is started. The steadily increasing numbers of international travellers stress the importance that worldwide, and not only in endemic countries, doctors become familiar with the spectrum of tropical or travel-related diseases.
